# Transmission of Human and Macaque *Plasmodium* spp. to Ex-Captive Orangutans in Kalimantan, Indonesia

**DOI:** 10.3201/eid1212.060191

**Published:** 2006-12

**Authors:** Michael J.C. Reid, Raul Ursic, Dawn Cooper, Hamed Nazzari, Melinda Griffiths, Birute M. Galdikas, Rosa M. Garriga, Mark Skinner, Carl Lowenberger

**Affiliations:** * Simon Fraser University, Burnaby, British Columbia, Canada;; † Orangutan Foundation International, Los Angeles, California, USA;; ‡ Orangutan Care Center and Quarantine, Desa Pasir Panjang, Kaliamantan Tengah, Indonesia

**Keywords:** Orangutans, *Pongo pygmaeus*, macaques, *Macaca* sp., malaria, *Plasmodium vivax*, *Plasmodium silvaticum*, *Plasmodium pitheci*, *Plasmodium cynomolgi*, cross-species transmission, research

## Abstract

Data are lacking on the specific diseases to which great apes are susceptible and the transmission dynamics and overall impact of these diseases. We examined the prevalence of *Plasmodium* spp. infections in semicaptive orangutans housed at the Orangutan Care Center and Quarantine, Central Kalimantan, Indonesia, by using a combination of microscopic and DNA molecular techniques to identify the *Plasmodium* spp. in each animal. Previous studies indicated 2 orangutan-specific *Plasmodium* spp., but our data show 4 *Plasmodium* spp. These findings provide evidence for *P. vivax* transmission between humans and orangutans and for *P. cynomolgi* transmission between macaques and orangutans. These data have potential implications for the conservation of orangutans and also for the bidirectional transmission of parasites between orangutans and humans visiting or living in the region.

## Abstract 2

We identified 4 discrete *Plasmodium* spp. sequences from the blood of orangutans, including 1 of *P. vivax*, which has implications for human residents and orangutan rehabilitation programs.

The following great apes are classified as endangered: the robust chimpanzees, Pan troglodytes; the gracile chimpanzee/bonobo, Pan paniscus; the gorilla, Gorilla gorilla; and the orangutans, Pongo pygmaeus and P. abelii. Habitat loss and hunting by humans are 2 direct threats to the survival of the great apes (*1*). Until recently, diseases have been overlooked as key threats to primate conservation efforts (*2*). However, recent research has emphasized the threat of disease transmission between human and nonhuman primates and the effects of these diseases on nonhuman primates (*3*). Malaria, caused by protozoan parasites in the genus Plasmodium, is 1 disease identified as a potential threat to the conservation of orangutans (*4*). Two species of Plasmodium naturally infect orangutans: P. pitheci, first isolated from the blood of a Bornean orangutan (*5*), and P. sylvaticum, identified from orangutans housed at the Sepilok Orangutan Rehabilitation Centre (SORC), Sabah, Malaysia (*6**,**7*). Both P. pitheci and P. silvaticum have tertian periodicities (*5**,**7*) and are distinguishable from human plasmodia (*7*). Three major studies of orangutan malaria at SORC (*4**,**5**,**7*) found prevalences of infection of >50%, which may have been influenced by the unusually high population density of orangutans at SORC, estimated at 100/km^2^ (*4*). Wolfe et al. (*4*) found the highest Plasmodium spp. prevalence at 93.5% (29/31) in captive animals but 11.6% (5/43) in wild orangutans.

Recent reports indicate that nonhuman primate plasmodia are the source of zoonotic disease outbreaks among humans in Thailand and Malaysia (*8**,**9*). Although this finding has implications for human disease outbreaks, few studies have investigated the distribution and transmission of Plasmodium spp. among orangutans and whether these great apes serve as reservoirs for human infections. Similarly, no studies have indicated that human plasmodia might infect and cause the death of captive or feral orangutans, a finding which would have serious implications for great ape conservation efforts. We report here the identification of plasmodia found in semicaptive and recently arrived orangutans at the Orangutan Care Center and Quarantine (OCC&Q) in the province of Central Kalimantan, Indonesia.

## Materials and Methods

### Study Site and Population

The OCC&Q is located in the village of Pasir Panjang, ≈5 km from the city of Pangkalan Bun in the province of Central Kalimantan, Indonesia. The OCC&Q was established by the Orangutan Foundation International in 1998 to serve as a hospital, orphanage, and rehabilitation center for sick and injured orangutans. Most orangutans are delivered by police or forestry officials after they have been confiscated from illegal pet owners; thus, the history of these animals is often unknown. All orangutans living at the OCC&Q are considered to be semicaptive; during the day, these orangutans have access to a nursery forest where they can learn the skills necessary for survival in the wild. At night these orangutans return to cages for sleep. Approximately 200 orangutans were housed at the OCC&Q during the 2003 study period. Samples were obtained from resident orangutans at OCC&Q as part of biannual health checks done by OCC&Q veterinary staff, and samples were obtained from newly confiscated animals as part of routine medical examinations. Orangutans were grouped into 4 categories on the basis of their residence history at OCC&Q: 1) OCC&Q residents (living at OCC&Q for >4 months) (n = 69), 2) newly confiscated arrivals (living at OCC&Q for <4 months) (n = 14), 3) newly confiscated arrivals with a previous history of treatment for malaria (n = 1), 4) newly arrived animals that had at 1 time been ex-captives, previously released back into the forest (n = 2). All animals also were grouped by size, which was used to estimate age: small (<15 kg), medium (15–30 kg), and large (>30 kg) (Table).

**Table:** Demographic data and infection status of orangutans housed at the Orangutan Care Center and Quarantine (OCC&Q), Central Kalimantan, Indonesia

Demographic category	Size*	No.	Sex		Blood smear positive for *Plasmodium* spp.		DNA positive for *Plasmodium* spp.	
			M	F	M	F	M	F
OCC&Q residents	Small	4	3	1	1	0	0	0
	Medium	61	28	33	7	6	3	1
	Large	4	3	1	0	0	0	0
Newly confiscated arrivals	Small	14	5	9	2	6	2	5
	Medium	0	0	0	0	0	0	0
	Large	0	0	0	0	0	0	0
Newly confiscated arrivals treated previously for malaria	Small	1	0	1	0	0	0	0
	Medium	0	0	0	0	0	0	0
	Large	0	0	0	0	0	0	0
Newly recaptured feral animals	Small	0	0	0	0	0	0	0
	Medium	2	2	0	2	0	2	0
	Large	0	0	0	0	0	0	0
Total	Small	19	8	11	3	6	2	5
	Medium	63	28	33	9	6	5	1
	Large	4	3	1	0	0	0	0

*Size classes were used as an estimate of age. Small orangutans weigh <15 kg, medium orangutans weigh 15–30 kg, and large orangutans weigh >30 kg.

### Sample Collection and Preservation

Blood samples were collected by an OCC&Q veterinarian with a 25-gauge × 1⅝-inch PrecisionGlide needle (Becton, Dickinson, and Company, Oakville, Ontario, Canada) and a 3-mL syringe. Some animals were sampled more than once so the effectiveness of antimalarial chemotherapy could be monitored. Thin and thick blood smears were prepared, fixed with methanol, stained with 10% Giemsa stain for 30 min, and destained with water (*10*). Samples were examined microscopically under 1,000× magnification, and levels of parasitemia were estimated (*10*). Aliquots of blood (5–10 μL) were placed by pipette onto each of 4 circular areas on Whatman FTA Classic Cards (Whatman Inc., Florham Park, NJ, USA), dried overnight at room temperature, and transported to Simon Fraser University for subsequent analysis.

### DNA Extraction

DNA was extracted from the Whatman FTA Classic Cards following the manufacturer's instructions (*11**,**12*). In subsequent PCR analyses, we used an entire punch or 2 μL of eluted DNA per reaction.

### PCR Analysis

We used primers designed against the 18S small subunit ribosomal RNA (*4**,**13*). The DNA samples were used in a 3-step PCR process. In step 1, PCR of DNA on the disks or from elutant was amplified with primers rPLU1 (5´-TCA AAG ATT AAG CCA TGC AAG TGA-3´) and rPLU5 (5´-CCT GTT GTT GCC TTA AAC TCC-3´) in a standard 50-μL PCR with a PTC-200 Thermocycler (MJ Research; Waltham, MA, USA) under the conditions of 94°C for 4 min, and 35 cycles at 94°C (30 sec), 55°C (1 min), 72°C (1 min), with an additional extension at 72°C (4 min).

In step 2, this product was used in a nested PCR with primers rPLU 3 (5´-TTT TTA TAA GGA TAA CTA CGG AAA AGC TGT-3´) and rPLU Cal 2 (5´-CGC TAT TGG AGC TGG AAT TAC C-3´) in a 25-μL reaction under the conditions of 94°C (4 min) and 35 cycles at 94°C (10 sec), 60°C (10 sec), 72°C (45 sec), with an additional extension at 72°C (4 min). PCR products were size fractionated by electrophoresis on a 1% agarose gel containing ethidium bromide and examined on a BioDoc gel documentation System (UVP, Upland, CA, USA). A ≈500-bp band confirmed Plasmodium spp. DNA in the initial PCR sample.

We used the DNA from step 1 that tested positive in step 2 in a third PCR with primers rPLU 3 (5´-TTT TTA TAA GGA TAA CTA CGG AAA AGC TGT-3´) and rPLU Cal (5´-ACA CAW RGT KCC TCT AAG AAG C-3´) by using BD Sprint Advantage Single Shots (Clontech, Palo Alto, CA, USA) under the conditions of 95°C (1 min), and 35 cycles at 95°C (30 sec), and 58° (3 min), with an additional extension at 58°C (3 min). These nested primers amplified an ≈1,500-bp fragment that contains 3–4 variable regions of the ribosomal sequence that can be used for species determination. After electrophoresis, PCR products were excised from the gel, purified by using a Qiagen Gel Purification Kit (Qiagen, Valencia, CA, USA), and ligated into pGEM-T-Easy Vector (Promega, Madison WI, USA). Putative transformants were identified by using blue-white screening of XL1-Blue Cells (Stratagene, La Jolla, CA, USA), grown overnight in 5 mL LB medium with ampicillin (100 μg/μL) and purified by using Wizard Plus Miniprep DNA Purification System (Promega). Sequencing of clones was done by using BigDye Chemistry (version 3.1) (Applied Biosystems, Foster City, CA, USA) and with the plasmid primers SP6 and T7 (*14*).

### Data Analysis

The nucleotide sequences obtained were compared with those Plasmodium spp. sequences available in public databases by using BLASTN (nucleotide-nucleotide) (available from http://www.ncbi.nlm.nih.gov/blast/). All available sequences of the Plasmodium spp. 18S sRNA gene, which contained complete sequences of our target region, were downloaded.

## Results

### Field Results

We collected 97 blood samples from 86 animals: 19 small, 63 medium, and 4 large orangutans, of which 41 were males and 45 were females (Table). Of the 69 OCC&Q residents tested (34 males, 35 females), 14 (20.3%) tested positive for Plasmodium spp. infections (8 males and 6 females) (Table). Eight of 14 newly arrived animals tested positive for Plasmodium spp. (57%) (2 small males and 6 small females) (Table). The newly arrived animal treated previously for malaria symptoms proved negative for Plasmodium spp. Both previously released ex-captive, medium-sized male orangutans, brought to OCC&Q from a release site in Tanjung Puting National Park for treatment of amebic dysentery, tested positive for Plasmodium spp. (Table).

### DNA Analysis

**Figure 1:**
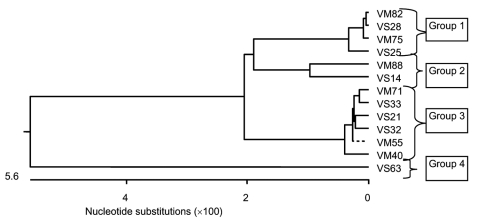
Phylogenetic tree of obtained sequences at the nucleotide level. Phylogenetic analysis of 14 individual sequences of the 18S small subunit ribosomal RNA gene (nucleotide level) isolated from the blood of orangutans housed at the Orangutan Care Center and Quarantine by PCR. Group 1 (*Plasmodium cynomolgi*–like) is represented by sequences from VS25, VS28, VM75, and VM82; group 2 (*P. inui*–like) is represented by sequences from VS14 and VM88; group 3 (*P. cynomolgi*) is represented by VS21, VS32, VS33, VM40, VM55, and VM71; and group 4 (*P. vivax*) is represented by sequence VS63.

We amplified, cloned, and sequenced a ≈1,500-bp segment of the Plasmodium spp. 18S sRNA gene from 13 of the 24 orangutans whose blood had been found positive for Plasmodium spp.: 4 from OCC&Q residents (3 males, 1 female), 7 from newly confiscated arrivals (2 males, 5 females), and 2 newly arrived feral animals (2 males) (Table). We aligned the sequences with similar sequences available in the databases, and generated phylogenetic trees based on a nearest neighbor analysis at the nucleotide level. We then aligned at the nucleotide level the sequences we obtained from our 13 Plasmodium spp.–infected orangutans. Phylogenetic trees showing the nearest neighbor relationships of our sequences were created from these alignments (Figure 1).

**Figure 2:**
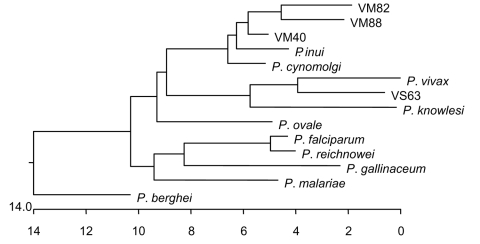
Phylogenetic tree of small subunit ribosomal RNA from different *Plasmodium* spp. Sequences were downloaded from GenBank, aligned by using CLUSTAL W (Megalign, DNA Star, Madison, WI, USA) and the tree generated by nearest-neighbor analysis. Once the sequences were aligned, we also aligned our representative sequences with the 2 nearest matches for more detailed determination of closest associations. Sequences used and their GenBank accession nos. were *P. gallinaceum* (M61723), *P. berghei (*AJ243513), *P. falciparum* (AL929354), *P. ovale* (AJ001527), *P. malariae* (AF88000), *P. vivax* (U03080), *P. cynomolgi* (L08241), *P. fragile* (M61722), *P. knowlesi* (U83876), *P. reichenowi* (Z25819), *P. simium* (U69605), and *P. inui* (U72541), clone 40 (*P. cynomolgi* [DQ660816]), clone 63 (*P. vivax* [DQ660817]), clone 82 (*P. cynomolgi*–like [DQ660818]), and clone 88 (*P. inui*–like [DQ660819]).

The 13 sequences we obtained form 4 distinct groups at the nucleotide level. On the basis of these groupings, we designated samples VS25, VS28, VM75, and VM82 as group 1; samples VS14 and VM88 as group 2; VS21, VS32, VS33, VM40, VM55, and VM71 as group 3; and VS63 as group 4. From each of these groupings, we selected 1 sequence to represent the entire group. These representative samples were then aligned at the nucleotide and translated amino acid level with available sequences downloaded from the databases to generate a phylogenetic tree (Figure 2).

Group 1 (representative sequence VM82) consists of 1,519 bp at the nucleotide level and translates to a putative protein with a length of 506 amino acids (aa). VM82 shares the greatest sequence identity with P. cynomolgi (94%) and P. inui (95%). A phylogenetic tree shows the close association with P. inui and P. cynomolgi. We then aligned VM82 solely with its 2 closest homologs, P. inui and P. cynomolgi. These 3 sequences are highly conserved but have variable areas between bases 106–155, 633–672, 720–730, and 1,018–1,050, which indicate a closer similarity with P. cynomolgi. Because of differences between VM82 and P. cynomolgi in the region of bases 143–155, 646–673, and 1,023–1,043, we designated this group as a P. cynomolgi–like parasite.

Group 2 (representative sequence VM88) consists of 1,544 bp at the nucleotide level and translates to a putative protein with a length of 514 aa. Throughout the variable regions, VM88 shares substantial similarity with P. hylobati (89%–96%), P. inui (90%–97%), P. fieldi, (89%–95%) and P. cynomolgi (90%–95%). We then aligned VM88 solely with its 2 closest homologs, P. inui and P. cynomolgi. These 3 sequences are highly conserved but show 2 large variable areas between bases 113–175 and 630–670. Despite sharing the greatest homology with P. inui, differences between VM88 and P. inui in the region of bases 110–175, 712–735, 789–887, and 1,495–1,551 indicate that this is a P. inui–like parasite.

Group 3 (representative sequence VM40) consists of 1,515 bp at the nucleotide level and translates to a putative protein with a length of 505 aa. VM40 shares significant identity with 3 Plasmodium spp. at the nucleotide level: P. hylobati, (96%–97%), P. inui (95%–97%), and P. cynomolgi (96%–98%). Alignment of VM40 solely with its 2 closest sequences indicated this sequence is homologous to P. cynomolgi.

Group 4, sequence VS63, consists of 1,582 bp at the nucleotide level and translates to a putative protein with a length of 526 aa. At the nucleotide level, VS63 shares the greatest identity with P. simium (97%–98%) and P. vivax (96%–98%).

## Discussion

Our results indicate that newly arrived orangutans to OCC&Q are statistically more likely to be infected with Plasmodium spp. than resident orangutans (χ^2^ = 8.11, degrees of freedom [df] = 1, p<0.01). Because these animals were confiscated from humans, we do not know their history or often the specific geographic region from which they originated. Increased levels of stress caused by time spent in direct contact with humans, reduced arboreality, and lack of a nutritious and normal diet may contribute to increased levels of infection (*4*).

Small orangutans were significantly more likely to be infected with Plasmodium spp. than medium orangutans (χ^2^ = 3.91, df = 1, p<0.05) or medium and large orangutans combined (χ^2^ = 4.59, df = 1, p<0.05). Whether these results are coincidental or whether older animals have some protection against reinfections with Plasmodium spp. has not been studied. In humans, most deaths attributed to malaria occur in young children, and evidence of age-acquired immunity against Plasmodium spp. has been found (*15*). However, such studies in other primates are few, and we can only speculate that the same phenomenon might be evident here.

We were unable to amplify Plasmodium spp. DNA from 9 of the orangutans identified in the field as positive. This may have been due to poor blood preservation, DNA degradation, infections with very low levels of parasitemia, or a misidentification of Plasmodium spp. in the initial blood smears.

No published data are available on the level of illness and death suffered by orangutans as a direct result of malaria, nor do data exist on the ability of the orangutan immune response to clear these infections. As a result, the orangutan plasmodia have been considered benign. However, orangutan rehabilitation facilities in Sumatra and Kalimantan have reported the elimination of debilitating, malarialike symptoms after treatment with antimalarial drugs ([16]; C. van Schaik, pers. comm., 2002). In 2002, OCC&Q treated malarialike symptoms in several orangutans with antimalarial drugs. These animals responded to treatment and returned to normal activity, but this does not definitively prove that their illnesses were the result of malaria (*16*). Because of the paucity of studies on malaria in orangutans, all done at SORC (*4**,**7*), we can only compare our data gathered at OCC&Q with data on the semicaptive orangutans tested at SORC (*4*). However, that study found a high prevalence of infection (93.5%) compared with our study (20.3%). Wolfe et al. (*4*) suggest that several behavioral and ecologic factors may contribute to higher rates of infection with Plasmodium spp. among semicaptive orangutans such as decreased arboreality, decreased day ranges, changes in social structure, increased population density, dietary changes, and stress. Each of these factors is also found to some degree in the orangutans housed at OCC&Q. Many ecologic factors differ between these 2 study sites. The region around the OCC&Q is primarily peat swamp forest (*17**,**18*), which typically does not support the growth and development of mosquito larvae. Wolfe et al. (*4*) also discussed the potential role of human activities that contributed to the high prevalence of Plasmodium spp. at SORC. These included the proximity of SORC to human settlements, very high densities of orangutans, and the effects of human-made structures such as drainage ditches, which increase the availability of standing water and vector mosquito population. However, no data exist on what mosquito species inhabit these regions or which species might transmit nonhuman plasmodia to orangutans.

Our DNA analyses show 4 distinct groups of Plasmodium spp. in the orangutans housed at OCC&Q, on the basis of an ≈1,500-bp segment of the 18S sRNA, considered an "ideal target" for Plasmodium spp. identification (*19*). Previous studies that relied on parasite morphology found 2 plasmodia in orangutans; P. pitheci and P. silvaticum (*4**,**7*), but no DNA sequences with which we could compare our data are available in the databanks for these 2 species. The macaque parasite P. inui has also been reported in orangutans housed at SORC (*4*), but these data have yet to be published. Two of our 4 sequences (groups 1 and 2) may possibly represent P. silvaticum and P. pitheci sequences. These are members of the P. vivax/Southeast Asian primate Plasmodium spp. group of parasites as are the macaque plasmodia P. cynomolgi, P. inui, P. fragile, and P. knowlesi (*20**–**26*).

The group 3 sequences align best with P. cynomolgi, a macaque Plasmodium sp. that can infect humans. Plasmodium cynomolgi, first identified in Javan Macaca fascicularis (*5*), is also a member of the P. vivax/Southeast Asian primate plasmodia group (*20**–**26*) and has been studied as the primate counterpart to human P. vivax (*27*). The presence of this parasite in orangutans indicates the cross-species transfer of Plasmodium spp. between macaques and orangutans. Macaques are common throughout Kalimantan and often can be seen crossing the road into and out of the nursery forest used by orphaned orangutans housed at OCC&Q. Although no confirmed reports exist of orangutans being infected with plasmodia specific to other primates, humans have been infected naturally with at least 2 species of macaque plasmodia (*5**,**8**,**9*). A macaque parasite, P. knowlesi, naturally infects humans in Malaysia (*5*) and continues to be an important zoonotic infection in Southeast Asia (*8**,**9*). Finding 1 of these species in orangutans housed at OCC&Q would not be surprising.

The group 4 sequence is the most relevant. This sequence aligns most closely with P. simium (found only in the New World) and P. vivax (found throughout Southeast Asia) and secondarily with P. knowlesi (Figure 2). When we compared several P. knowlesi sequences with each other, with VS63, and with P. vivax, the latter 2 were essentially identical, and BLAST pairwise comparison generated 1 contiguous sequence with >98% shared identity. Areas of notable differences were found between VS63 and P. knowlesi. The BLAST pairwise comparison indicates 5 discrete regions of alignment separated by nonsimilar regions, which indicates that VS63 is not a known variant of P. knowlesi.

P. vivax and P. simium are genetically indistinguishable at 13 microsatellite loci and 8 tandem repeats (*27*). Because P. simium does not exist in Southeast Asia, we have the first report of an orangutan being infected with human P. vivax. This orangutan was a recent arrival at OCC&Q, had been confiscated from human captivity, had extensive interactions with humans in a domestic setting, and had arrived at OCC&Q with a low-level infection that was untreated 3 months before this study began, which indicates a fully functional infection.

P. vivax is one of the most widespread of the human plasmodia; it infects 70–80 million persons in the low-lying, coastal, and marshy regions of the world (*5**,**24*). Data on the human plasmodia of Central Kalimantan are not easily accessible through the available scientific databases. We assume that P. vivax is present in this region on the basis of the results of our study, the widespread nature of this parasite (*5**,**24*), and reports of chloroquine-resistant P. vivax in neighboring West Kalimantan (*28*). That P. vivax is infective to orangutans is not surprising. Current evidence suggests that P. vivax originated 80,000–10,000 years ago from a macaque Plasmodium sp (*22**–**24*). Because humans are genetically closer to orangutans than to macaques, if P. vivax arose as the result of a recent host switch, then orangutans also could be infected with P. vivax.

Our data indicating that orangutans can be infected with human P. vivax, and the corresponding infection of humans with macaque plasmodia (*8**,**9*) emphasize the potential importance of the bidirectional transmission of these parasites between humans and nonhuman primates living in close proximity. Increasing our understanding of potential host species and phylogenetic associations of closely related parasites may help identify the origins of human diseases (*3**,**29**,**30*).

The data presented here suggest that Bornean orangutans (Pongo pygmaeus) may be infected by 4 species of plasmodia; 2 of these may represent the previously identified orangutan plasmodia P. pitheci and P. silvaticum. Macaque malaria in orangutans suggests cross-species transmission of a parasite between macaques and orangutans living sympatrically in Kalimantan (as had been described for human infections with macaque malaria in Thailand and Malaysia) (*8**,**9*). Orangutans also may be susceptible and may be exposed to infection from the human parasite, P. vivax, although few data are available on the symptoms of macaque or human malaria infections in orangutans. Nonetheless, these findings could have important implications for orangutan conservation and rehabilitation programs and for humans who live in close proximity to orangutans. The role of humans and great apes as reservoirs of parasites that can be shared and transmitted between both hosts has not been well-studied. Conservation and rehabilitation programs that permit visits by humans must take into consideration the exchange of parasites between humans and endangered species, the implications of human parasites on the survival of the great apes in these centers, and the potential of these animals to serve as reservoirs of human parasites.
